# The Client Empowerment in Community Health Systems Scale: Development and validation in three countries

**DOI:** 10.7189/jogh.11.07010

**Published:** 2021-03-10

**Authors:** Tracy L McClair, Pooja Sripad, Alain Casseus, Sharif Hossain, Timothy Abuya, Ann Gottert

**Affiliations:** 1 Population Council, Washington, D.C., USA; 2 Zanmi Lasante, Mirebalais, Haiti; 3 Population Council, Dhaka, Bangladesh; 4 Population Council, Nairobi, Kenya

## Abstract

**Background:**

Effectively measuring client empowerment is critical for monitoring and supporting empowerment through interventions, including via community health workers (CHWs) on the front line. Yet a comprehensive measure capturing the multidimensional aspects of client empowerment is not currently available. We aimed to develop and validate the Client Empowerment in Community Health Systems (CE-CHS) Scale in three countries.

**Methods:**

We used data from cross-sectional surveys from 2019-2020 with clients of CHWs in Bangladesh (n = 1384), Haiti (n = 616), and Kenya (n = 306). Nineteen candidate CE-CHS Scale items were adapted from existing health empowerment and sociopolitical control scales. Items spanned three hypothesized sub-domains: personal agency around health (eg, “I feel in control of my health”), agency in sharing health information with others (eg, “I feel confident sharing health information with my family/friends”), and empowerment in community health systems (eg, “Most facility/managers would listen to any concerns I raise”). Face and content validity of items were assessed via two focus group discussions in Haiti. For each country, we conducted split-sample exploratory/confirmatory factor analyses (EFA/CFA) and assessed internal consistency reliability. We assessed convergent validity by comparing final full-scale and sub-dimension scores to theoretically related variables.

**Results:**

All participants in Bangladesh and Kenya were female, as were 85% in Haiti. Mean age in Bangladesh and Kenya was around 25 years; 40 in Haiti. EFA/CFA resulted in a final 16-item CE-CHS Scale representing the three hypothesized sub-scales. Three items were dropped in EFA due to poor performance. CFA fit statistics were good for the full-scale and each sub-scale. The mean CE-CHS score (range 1 to 4) was 2.4 in in Bangladesh, 2.8 in Haiti, and 3.0 in Kenya. Cronbach’s alpha and ordinal theta of the full and sub-scales were greater than 0.7. Increased empowerment was associated with increased trust in CHWs, influence of CHWs on empowerment, satisfaction with CHW services, number of CHW interactions, civic engagement, and education, with slight variations in magnitude and significance by country.

**Conclusions:**

Findings suggest that the 16-item CE-CHS Scale is valid and reliable. This scale can be used to assess levels and determinants of, and changes in, client empowerment in future implementation research and monitoring of community health systems.

Empowered community members are more likely to engage in behaviors that promote their own health and that of their families and communities [1]. Client empowerment in a community health system, an extension of the broader primary health care system, can be influenced by community health workers (CHWs). Agarwal et al. define client empowerment as “community members’ agency – awareness of and access to – community health services as well as the participation or engagement of communities in shaping and maintaining community health services (including CHW programs) [2].”

Client empowerment is both a process that can contribute to improved health outcomes, and can be conceptualized as an important outcome itself [3]. Previous research has shown that individuals can better achieve their own health goals if they are empowered to engage with others in their community who are in similar circumstances as them [1]. For example, participatory learning exercises in women’s groups, often facilitated by CHWs, have been shown to be associated with maternity care seeking and reductions in neonatal and maternal mortality [4-7].

Empowerment is a multidimensional and highly contextual concept that can occur at multiple levels of the socioecological model, which posits that the entire ecological system, composed of four levels – microsystem, mesosystem, exosystem, and macrosystem – is necessary for understanding human development [8]. In the context of community health systems, at the individual (microsystem) level, clients can be empowered to have personal agency around their own and/or their family’s health. At the interpersonal (mesosystem) level, clients can be empowered to share health information with their friends and family. At the community (exosystem) level, clients can engage with community health systems at large and exert sociopolitical control – the belief of one’s skills and capabilities in the context of social and political systems such as community health systems [9]. Client empowerment can also depend on societal values inherent in countries’ unique sociocultural contexts (macrosystem).

CHWs are essential to health systems since they provide a basic level of primary care conveniently within the community while linking community members with health institutions [10]. CHWs are uniquely positioned to support universal health coverage goals by reaching underserved populations who may have limited access to health services with health promotion and preventative services, for example, in the areas of family planning (FP), maternal and child health, nutrition, HIV, malaria, and tuberculosis [11]. CHWs also play an important role in meeting clients’ psychosocial needs by being accessible in their communities, serving as an advocate for health and well-being, and listening and helping address clients’ health concerns. Given the role of CHWs in their communities, they can support client empowerment in the context of community health systems.

Empowerment of community members has been recognized as an essential component of community health systems. The first two of ten principles to institutionalizing community health outlined by Pfaffmann Zumbruni et al. center around empowerment: empower communities to build community health systems and hold them accountable [12]. However, the authors are not aware of a previously validated, comprehensive empowerment scale that captures the multi-level aspects of client empowerment in the context of community health systems, which could also be important for assessing and improving CHW performance. Notably, many existing empowerment scales largely focuses on empowerment at the individual level, such as psychological empowerment, while less attention is paid to empowerment at the interpersonal and community levels, which are necessary for the sustainability of positive health behaviors and outcomes [13-17].

Under the Frontline Health project – a three-and-a-half year initiative led by Population Council to harmonize CHW performance metrics and promote the use of evidence in CHW programming – this study was conducted to develop and validate the Client Empowerment in Community Health Systems Scale (CE-CHS) in three countries [18].

## METHODS

### Study design and setting

We employed a scale development approach by generating items through a review of the literature on health agency and sociopolitical empowerment scales and domains, conducting qualitative research around the conceptualization of client empowerment in community health systems, and engaging in expert consultation to assess face validity [19].

The resulting nineteen candidate scale items were embedded in cross-sectional surveys of clients of CHWs. Surveys were part of larger studies in Bangladesh, Haiti, and Kenya that evaluated country-specific implementation research questions around CHW programs [18]. The health topic areas of focus for these studies were different in the three settings (FP in Bangladesh, general health in Haiti, and antenatal/postnatal care in Kenya) corresponding to the varied topics of CHW’s work globally.

### Study participants and sampling

Our sample included clients who had recently interacted with a CHW. In Bangladesh, this included women ages 15 to 35 years who were non-users of any FP method or who discontinued methods in the last 3 months and who had interacted with Family Welfare Assistants (FWAs) or Family Welfare Visitors (FWVs) in the last six months in 12 unions in Keraniganj upazila (sub-district), Dhaka. In Haiti, the sample included women and men ages 15 and older who interacted with Agents de Santé Communautaire Polyvalent (ASCPs) in the last six months on any health issue in three communes in Artibonite and Centre departments. In Kenya, the sample included pregnant women in the second or third trimester and mothers of babies 0-59 days ages 16 and older who saw a community health volunteer (CHV) for antenatal or postnatal counseling in the last three months in four sub-counties in Bungoma and Kilifi counties. Detailed descriptions of sampling strategies for Bangladesh and Kenya are described elsewhere [20,21]. In Haiti, a simple random sample approach of the combined ASCP registry and digital database was used to sample clients recruited by Zanmi Lasante’s team and a community guide.

### Survey Procedures

All data collectors were extensively trained and obtained informed consent from all participants by signature or thumb print for those with limited literacy. Using relevant languages (Bengali in Bangladesh, Creole in Haiti, and Kiswahili in Kenya), data collectors administered the survey in the clients’ home in Bangladesh and Haiti and in health facilities in Kenya. Surveys were paper-based in Bangladesh and tablet-based using Open Data Kit software in Haiti and Kenya. Data from all three countries were collected between July 2019 and January 2020.

Ethical approval was obtained from the Population Council Institutional Review Board in New York, USA (*p874, p879, and p876)*, as well as the Bangladesh Medical Research Council (20608052019), the Zanmi Lasante Institutional Review Board in Haiti (*ZLIRB2732019)*, and AMREF Health Africa Ethics and Social Review Committee in Kenya (*p573)*.

### Scale development

A pool of testable empowerment scale items was adapted from previously published scales as well as qualitative research conducted in Haiti. We undertook an iterative process by drafting questions with local study team members and experts in scale development and community health [2,22-24]. We drew on Bann et al.’s empowerment scale to develop the personal agency sub-scale [25]. Findings from the Population Council studies under the Ending Eclampsia project on the health and well-being benefits of community health groups informed the development of the information sharing sub-scale [26]. We drew from Peterson et al.’s sociopolitical control scale to develop the engagement with community health systems sub-scale [9].

Nineteen testable items fell broadly under three hypothesized domains corresponding to the individual, interpersonal, and community levels of the socioecological model: personal agency around health, agency in sharing health information with others, and engagement in community health systems. The scale was introduced by the statement “Now I will read a series of statements. Please let me know how much you agree or disagree with each of these statements.” Respondents were then asked whether they ‘strongly disagree’, ‘disagree’, ‘agree’, or ‘strongly agree’ with each statement.

All scale items were translated into Bengali, Creole, or Kiswahili, and checked for meaning and comprehension by the study team, and items were pretested to refine item wording. To assess face and content validity of the preliminary set of items, two focus group discussions were held in Haiti with men (n = 6) and women (n = 7) who met survey eligibility criteria. They were selected purposively by Zanmi Lasante staff and CHW supervisors. After participants self-administered the set of items, participants re-reviewed items together one by one, and for each discussed: “What does this statement mean to you?’, ‘Is this a useful/relevant statement?’, ‘Does the language used make sense?’, ‘How can it be changed?’, ‘Should we keep or remove it?’ There were no issues with comprehension of any of the scale items and all items translated well into the local language. Further, all items were deemed important. Based on this discussion, all 19 items were retained.

### Data analysis

All statistical analyses were performed using Stata v15 (StataCorp LLC, College Station TX, USA). We began by exploring response distributions for each item to confirm whether each had adequate variation (eg, did not have >90% in one category) to proceed to psychometric analyses.

Next, we randomly split each country’s sample, designating half for exploratory factor analysis (EFA) and half for confirmatory factor analysis (CFA). All empowerment items were scored 1 to 4, ‘strongly disagree’ to ‘strongly agree.’

For EFAs, we conducted principal components analysis (PCA) to explore the number of factors underlying the set of items, by assessing the number of eigenvalues over 1.00, generating scree plots, and conducting parallel analysis to gauge the number of factors above the ‘elbow’ (for scree plot) or line (for parallel analysis) [19]. Items were retained for testing in the CFA model if they had low uniqueness (<0.5), adequate factor loadings (>0.3), and the item was conceptually necessary based on face and content validity.

To test the emergent factor structure from the EFA, we conducted CFA using the CFA samples for each country. We retained items with statistically significant factor loadings (*P* < 0.05). We then assessed CFA model fit using common cutoff criteria including the root mean square error of approximation (RMSEA) of <0.10 (ideally <0.05), comparative fit index (CFI) and the Tucker-Lewis index (TLI) of >0.90 (ideally >0.95), and standardized root mean square residual (SRMR) of <0.08. We incorporated modification indices into CFA structural equation models to remove any correlated error terms due to similarly worded items, then reassessed model fit. We assessed model fit for each of the three sub-scales as well as the full (multidimensional) scale.

For the set of items resulting from the EFA and CFA, we assessed internal consistency reliability using Cronbach’s alpha and ordinal theta; the latter assumes polychoric correlation and thus is more appropriate for items with limited response categories [27]. A cutoff of ≥0.7 was used to determine adequate internal consistency.

Finally, we assessed convergent validity using the full sample for each country. We used multivariate linear regression to explore associations between the full-scale scores, as well as sub-scale scores, with theoretically correlated variables including trust in CHWs, perceived influence of CHWs on empowerment, satisfaction with CHW services, number of CHW visits, client’s civic engagement, and education. Multivariate models were used to adjust for sociodemographic factors which could also contribute to differences in empowerment, including age, education, wealth, geographic area, parity (Bangladesh only), and sex (Haiti only). Analyses were also adjusted to account for clustering based on the sampling strategy in which CHWs listed potential study participants in their catchment areas in Bangladesh and Kenya. **Table 1** includes measures for sociodemographic characteristics and variables used to assess convergent validity.

**Table 1:** Measures for sociodemographic characteristics and variables used to assess convergent validity

Variable name	Variable type	Definition	
**Sociodemographic characteristics:**		
Age	Continuous	Respondents’ age in years at time of survey	
Education*	Categorical	Three categories including none, primary, secondary and above. In Haiti and Kenya, highest level of education/schooling attended; in Bangladesh, highest level of education completed	
Wealth	Categorical	Wealth quintiles based on a wealth index tailored for each country with questions such as “What is the main material of the floor of your dwelling?” with response options ‘cement’, ‘earth/sand’, ‘other’ [28]	
Geographic area	Categorical	12 unions in Bangladesh (Konda, Taghoria, Shuvadda, Sakta, Taranagar, Rohitpur, Kalatia, Hozratpur, Aganagar, Zingira, Basta, Kalindi); 3 communes in Haiti (Mirebalais, Verrettes, Petite Rivière de l'Artibonite); 4 sub-counties in Kenya (Kaloleni, Torgaren, Webuye West, Kilifi North)	
Sex	Binary	Male, female (in Haiti only, since all CHW respondents in Bangladesh and Kenya were female)	
Parity	Continuous	Number of children who live and do not live with the respondent (data available in Bangladesh only)	
**Convergent validity measures:**		
Number of CHW interactions	Categorical	Three categories including one time, two to three times, four or more times. In Haiti, number of CHW interactions in last six months. In Bangladesh, in last six months; note that for those who said 0 times (n = 367), it is assumed they saw a FWV, a facility-linked community health provider, and thus were included in the analysis, since questionnaire was a CHW exit interview. In Kenya half the sample is from ANC: “How many visits/contacts have you had with CHWs in the last three months?” and half the sample from PNC: “How many times have you been in contact with a CHW since childbirth?”	
Civic engagement	Continuous	Mean score on seven-item Civic Engagement Scale, with items such as “I like to work on solving a problem in my community rather than waiting for someone else to address it.” Four response options on Likert scale, ‘strongly disagree’, ‘disagree’, ‘agree’, ‘strongly agree’, ranging from 1-4. Items were adapted from Peterson et al’s sociopolitical control scale [9]. We conducted split sample EFA/CFA. EFA results confirmed that civic engagement is a unidimensional construct. CFA fit statistics were good and Cronbach’s alpha and ordinal theta were 0.89 or higher for all three countries.	
Trust in CHWs	Continuous	Mean score on 10-item Trust in CHWs Scale, ranging from 1-4. Limited to those who had contact with a community-based CHW in the last six months (excludes n = 367 in Bangladesh who saw FWVs). Details on the development and validity of the Trust in CHWs Scale are documented in Sripad et al [29].	
Influence of CHWs on empowerment	Continuous	Mean score on five-item scale assessing CHW influence on various aspects of client empowerment in Bangladesh and Kenya, with the following items:	
		• I can better make decisions about my health and my children’s health because of my interactions with CHWs.	
		• I can better share health information with others because of my interactions with CHWs.	
		• I can better get the care I need from my clinic because of my interactions with CHWs.	
		• I can better improve my clinic and/or the health system because of my interactions with CHWs.	
		• I can better contribute to my community because of my interactions with CHWs.	
		There were four response options on a Likert scale, ‘strongly disagree’, ‘disagree’, ‘agree’, and ‘strongly agree’, with mean range from 1-4. The research team developed the items in this scale to explicitly assess CHW influence on empowerment. We conducted split sample EFA/CFA. EFA results confirmed that influence of CHWs on empowerment is a unidimensional construct; CFA statistics were good and alpha and ordinal theta were 0.75 and higher, suggesting good reliability. Further, we found that increasing CHW visits was significantly associated with increasing influence of CHWs on empowerment scores.	
		In Haiti, a similar 2-item index was used, for example “Do your interactions with your CHW improve your ability to make decisions about your health?” Response options were binary yes/no, with mean range from 0 to 1.	
Satisfied with CHW services	Binary	Responded ‘very satisfied’ to “At your most recent visit with the CHW, how satisfied were you with the services you received from the CHW?” vs all other responses (‘very dissatisfied’, ‘somewhat dissatisfied’, ‘somewhat satisfied’)	

CHW – community health worker, EFA/CFA – exploratory factor analysis/confirmatory factor analysis, FWV – family welfare visitor, ANC – antenatal care, PNC – postnatal care

*Also used for convergent validity.

## RESULTS

A total of 2306 community members were included in analyses in Bangladesh (n = 1384), Haiti (n = 616) and Kenya (n = 306). Less than 1% of prospective participants refused to participate. **Table 2** describes sociodemographic characteristics by country. All participants in Bangladesh and Kenya were female; 85% were female in Haiti. Mean age in Bangladesh and Kenya was around 25 years and in Haiti, 40 years. Education levels varied by country, with low proportions of those with no education in Bangladesh and Kenya (<10%) and 58% with no education in Haiti. The number of CHW interactions varied by country. The proportion of participants reporting four or more visits was 15%, 63%, and 19% in Bangladesh, Haiti, and Kenya, respectively.

**Table 2:** Sample sociodemographic characteristics by country

	Bangladesh (n = 1384)	Haiti (n = 616)	Kenya (n = 306)
**n (%)**	**n (%)**	**n (%)**
Sex:			
Female	1384 (100.0)	524 (85.1)	306 (100.0)
Male	0 (0.0)	92 (14.9)	0 (0.0)
Age (mean, standard deviation) (range)	24.3 ± 5.1 (15-35)	40.0 ± 17.6 (15-90)	26.5 ± 6.1 (16-45)
Education:			
None	75 (5.4)	359 (58.3)	25 (8.2)
Primary	439 (31.7)	157 (25.5)	163 (53.3)
Secondary and above	870 (62.9)	100 (16.2)	118 (38.6)
Parity (mean, standard deviation) (range)	1.7 ± 0.8 (0-5)	n/a	n/a

For the split-sample EFA in Bangladesh (n = 692), Haiti (n = 308), and Kenya (n = 153), PCA, scree plot, and parallel analysis suggested 2-3 factors for all three countries. Responses were not normally distributed for all items, thus we used iterative principal factor specification. Further, we used promax rotation as factors were correlated. We removed three items based on EFA results: one item had high uniqueness (0.8) and two items did not load greater than 0.3 on any factor. The item with high uniqueness was: “I feel confident that I will be seen by a health provider at a facility”. This item was also deemed as not conceptually aligned with empowerment, and as likely depending more on facility capacity. The two items that did not load on any factor were: “I understand the important health issues that affect my community” and “I feel confident that I can ask questions to health providers at facilities”; it is possible respondents may feel the latter item is not relevant to them if in fact they did not have questions.

The EFA suggested a three-factor solution of 16 items total: 7 items representing *Personal agency around health*, 4 items representing *Agency in sharing health information with others*, and 5 items representing *Engagement in community health systems*. **Table 3** includes disaggregation of responses for each item in the resulting empowerment scale. Responses tended to skew towards ‘agree’ and ‘strongly agree’ for the 7-item *Personal agency around health* sub-scale, particularly in Bangladesh and Kenya. The 4-item *Agency in sharing-health information* sub-scale had more variation in item responses in all three countries compared to the *Personal agency around health* sub-scale. For the 5-item *Engagement in community health systems* sub-scale, in Bangladesh item responses skewed towards ‘strongly disagree’ and ‘disagree’, while responses were more varied in Haiti and Kenya. All factor loadings for the sub-scales and full-scale were significant (*P* < 0.001) and were greater than 0.3 in CFA (**Table 3**).

**Table 3:** CE-CHS Scale item frequencies, summary scores, and CFA factor loadings by country*

	Bangladesh (n = 1384)						Haiti (n = 616)						Kenya (n = 306)					
	**Strongly disagree, %**	**Disagree, %**	**Agree %**	**Strongly agree, %**	***CFA factor loadings***		**Strongly disagree, %**	**Disagree, %**	**Agree %**	**Strongly agree, %**	**CFA factor loadings**		**Strongly disagree** **%**	**Disagree, %**	**Agree %**	**Strongly agree, %**	**CFA factor loadings**	
**Personal agency around health**																		
I feel in control of my health.	0.3	2.2	62.1	35.5	*0.552*		2.8	28.6	67.9	0.8	0.680		1.3	2.3	52.3	44.1	*0.579*	
I know what to do when I have a health problem.	0.3	1.5	59.8	38.4	*0.731*		2.4	17.2	78.6	1.8	*0.817*		1.0	5.2	52.6	41.2	*0.825*	
I know what to do when there is a health problem in my family.	0.5	4.0	63.8	31.7	*0.793*		2.4	15.6	80.0	2.0	*0.917*		0.3	7.8	54.3	37.6	*0.831*	
I believe that I can participate in finding a solution to my health problem.	0.4	5.2	65.3	29.2	*0.780*		1.8	16.1	79.9	2.3	*0.894*		1.0	3.6	61.4	44.0	*0.803*	
When a health problem arises in my family, I’m able to help find a solution.	1.0	9.8	63.0	26.2	*0.801*		2.0	15.8	78.6	3.7	*0.849*		0.3	6.5	60.1	33.0	*0.838*	
When I have a health problem, I advocate for myself to make sure I get good care.	0.3	1.8	57.7	40.3	*0.661*		2.1	13.8	80.4	3.7	*0.861*		0.0	2.9	59.8	37.3	*0.752*	
When there is a health problem in my family, I advocate for them to get good care.†	0.7	5.3	63.5	30.5	*0.725*		2.1	14.2	80.4	3.3	*0.804*		0.3	4.6	59.2	36.0	*0.783*	
*Sub-scale mean (standard deviation) and higher order CFA loading*	3.28 (0.43)				*0.642*		2.81 (0.43)				*0.764*		3.32 (0.48)				*0.314*	
**Agency in sharing health information with others:**																		
I feel confident sharing health information with my family/friends in one-on-one conversations.	3.6	17.1	63.3	16.0	*0.389*		1.5	16.1	79.4	3.1	*0.773*		2.6	6.2	60.5	30.7	*0.384*	
I feel confident sharing health information with my family/friends when we are in a group.	9.6	29.6	50.9	9.8	*0.498*		1.6	16.1	77.8	4.6	*0.870*		3.6	15.4	57.2	23.9	*0.503*	
I feel confident sharing health information with others in my community in one-on-one conversations.	28.4	47.0	22.6	2.0	*0.918*		2.4	16.6	77.6	3.4	*0.958*		8.5	12.8	60.8	18.0	*0.819*	
I feel confident sharing health information with others in my community when in group/public settings.	38.9	48.6	11.1	1.5	*0.771*		2.3	18.3	75.5	3.9	*0.887*		11.8	25.2	60.7	12.4	*0.959*	
*Sub-scale mean (standard deviation) and higher order CFA loading*	2.32 (0.56)				*0.919*		2.83 (0.46)				*0.821*		2.93 (0.62)				*0.742*	
**Engagement in community health systems**																		
There are ways for me to participate in sharing my concerns/giving feedback to providers/managers at health facilities.	45.5	45.8	7.7	1.1	*0.702*		2.1	19.3	74.4	4.2	*0.822*		9.5	20.9	57.2	12.4	*0.754*	
There are ways for me to participate in sharing my concerns/giving feedback to local leaders in my community.	53.1	42.9	3.5	0.6	*0.916*		3.1	30.4	63.0	3.6	*0.878*		12.1	20.6	58.2	9.2	*0.767*	
Most facility providers/managers would listen to any concerns I raise.	47.1	45.4	6.9	0.7	*0.805*		2.6	14.8	78.1	4.6	*0.729*		3.6	9.5	68.0	19.0	*0.405*	
Most local leaders in my community would listen to any concerns I raise.	53.3	42.4	3.9	0.4	*0.889*		4.6	28.3	64.6	2.6	*0.913*		10.1	19.3	60.5	10.1	*0.635*	
I am able to participate in making decisions for my community.	59.0	38.7	1.8	0.5	*0.547*		2.6	25.3	66.1	6.0	*0.667*		14.4	16.0	59.8	9.8	*0.731*	
*Sub-scale mean (standard deviation) and higher order CFA loading*	1.54 (0.51)				*0.336*		2.75 (0.47)				*0.715*		2.75 (0.54)				*0.867*	
**CE-CHS score mean (standard deviation) (range 1-4)**	2.38 (0.37)						2.79 (0.39)						3.00 (0.41)					

CE-CHS – Client Empowerment in Community Health Systems, CFA – confirmatory factor analysis

*All CFA factor loadings significant at the *P* < 0.001 level. CFA sample sizes were n = 692, n = 308, and n = 153 in Bangladesh, Haiti, and Kenya, respectively. Percentages may not add up to exactly 100% due to rounding to one decimal place for ease of presentation.

†Missing 4 observations in Haiti.

After adding modification indices, goodness of fit statistics across the structural equation models were acceptable, with RMSEAs less than 0.1 (in some cases, less than 0.05), CFIs and TLIs were greater than 0.9 (in some cases greater than 0.95), and SRMR less than 0.01 (and in some cases less than 0.05) (**Table 4**). In Kenya, the RMSEA and TLI for the *Agency in sharing health information with others* sub-scale did not meet cutoff criteria (at 0.287 and 0.739, respectively). Higher-order CFA fit statistics were all significant, and sub-scale factor loadings on the higher-order factor were above 0.3 in Bangladesh and Kenya and 0.7 in Haiti (**Table 3**).

**Table 4:** Confirmatory factor analysis fit statistics by country*

	Bangladesh	Haiti	Kenya
**CE-CHS Scale:**			
RMSEA	0.048	0.063	0.016
CFI	0.979	0.971	0.998
TLI	0.969	0.954	0.997
SRMR	0.038	0.048	0.069
**Sub-scales:**			
Personal agency around health (7 items):			
RMSEA	0.085	0.089	0.071
CFI	0.986	0.990	0.987
TLI	0.958	0.976	0.977
SRMR	0.025	0.024	0.035
Agency in sharing health information with others (4 items):			
RMSEA	0.083	0.043	0.287
CFI	0.995	1.000	0.956
TLI	0.973	0.997	0.739
SRMR	0.013	0.005	0.033
Engagement in community health systems (5 items):			
RMSEA	0.056	0.057	0.092
CFI	0.999	0.996	0.971
TLI	0.990	0.989	0.941
SRMR	0.008	0.016	0.037

CE-CHS – Client Empowerment in Community Health Systems, RMSEA – root mean square error of approximation, CFI – comparative fit index, TLI – Tucker-Lewis index, SRMR – standardized root mean square residual

*Number of correlated errors: Full scale (Bangladesh: 8; Haiti: 17; Kenya: 18), *Personal agency around health* sub-scale (Bangladesh: 8; Haiti: 5; Kenya: 2); *Agency in sharing health information with others* sub-scale (Bangladesh: 1; Haiti: 1; Kenya: 1); *Engagement in community health systems* sub-scale (Bangladesh: 3; Haiti: 0; Kenya: 0).

Internal consistency reliability for the full scale and each sub-scale, assessed using Cronbach’s alpha and ordinal theta, were all at least adequate, at >0.7, and most above 0.8 or 0.9 (**Table 5**).

**Table 5:** Reliability of CE-CHS Scale and sub-scales

	Bangladesh (n = 1384)		Haiti (n = 616)		Kenya (n = 306)	
	**Alpha**	**Ordinal theta**	**Alpha**	**Ordinal theta**	**Alpha**	**Ordinal theta**
CE-CHS Scale (16 items)	0.860	0.900	0.912	0.967	0.856	0.915
Sub-scales:						
Personal agency around health (7 items)	0.885	0.930	0.941	0.970	0.913	0.954
Agency in sharing health information with others (4 items)	0.758	0.811	0.930	0.948	0.821	0.865
Engagement in community health systems (5 items)	0.888	0.929	0.829	0.925	0.732	0.771

CE-CHS – Client Empowerment in Community Health Systems

Mean CE-CHS score (range 1 to 4) was 2.38 in in Bangladesh, 2.79 in Haiti, and 3.0 in Kenya (**Table 2**). Mean *Personal agency around health* sub-scale score was 3.28, 2.81, and 3.32 in Bangladesh, Haiti, and Kenya, respectively. Mean *Agency in sharing health-information* was 2.32, 2.83, and 2.93 in Bangladesh, Haiti, and Kenya, respectively. Mean *Engagement in community health systems* was 1.54, 2.75, and 2.75 in Bangladesh, Haiti, and Kenya, respectively.

Descriptive statistics for the variables used to assess convergent validity are presented in **Table 6**. Mean civic engagement on a scale of 1 to 4 was 1.53 in Bangladesh and 2.66 in Haiti and Kenya. Mean influence of CHWs on empowerment on a scale of 1 to 4 in Bangladesh and Kenya was 2.18 and 3.18, respectively. Mean influence of CHWs on empowerment on a scale of 0 to 1 in Haiti was 0.91. Satisfaction with CHW services varied by country: 29%, 47%, and 93% were very satisfied in Bangladesh, Kenya, and Haiti, respectively.

**Table 6:** Frequencies of convergent validity items by country

	Bangladesh (n = 1384)	Haiti (n = 616)	Kenya (n = 306)
**n (%)**	**n (%)**	**n (%)**
Number of CHW interactions:*			
1 time	595 (43.0)	68 (11.0)	91 (30.7)
2-3 times	577 (41.7)	159 (25.8)	147 (49.7)
4+ times	212 (15.3)	389 (63.2)	58 (19.6)
Civic engagement (range: 1-4) (mean, standard deviation)	1.51 ± 0.53	2.66 ± 0.51	2.64 ± 0.68
Trust in CHWs (range: 1-4) (mean, standard deviation)†	3.49 ± 0.45	3.79 ± 0.43	3.39 ± 0.65
Influence of CHWs on empowerment (mean, standard deviation)‡	2.18 ± 0.55	0.91 ± 0.25	3.18 ± 0.58
Satisfied with CHW services	403 (29.1)	572 (92.9)	144 (47.1)

CHW – community health worker

*Missing 10 observations in Kenya.

**†**Limited to only those who had contact with a CHW in the last 6 months, since Trust in CHWs Scale specifically related to a CHW visit in the last 6 months; Bangladesh, n = 1017.

‡Range is 1-4 in Bangladesh and Kenya. In Haiti, range is 0 to 1 since mean score is reported from two yes/no questions.

In multivariate analyses adjusting for age, education, wealth, geographic location, parity (Bangladesh only), sex (Haiti only), and CHW clustering (Bangladesh and Kenya only), higher empowerment scores were consistently associated with higher levels of each of these related constructs, as hypothesized, although the magnitudes of associations varied (**Table 7**). Similar associations were seen for both the full CE-CHS and each of the sub-scales.

**Table 7:** Multivariate analysis of associations between CE-CHS Scale/sub-scales and related constructs (convergent validity)*†

	Bangladesh (n = 1384)				Haiti (n = 616)				Kenya (n = 306)			
	**CE-CHS**	**Personal agency around health**	**Agency in sharing health information with others**	**Engagement in CH systems**	**CE-CHS**	**Personal agency around health**	**Agency in sharing health information with others**	**Engagement in CH systems**	**CE-CHS**	**Personal agency around health**	**Agency in sharing health information with others**	**Engagement in CH systems**
Trust in CHWs‡	0.08^d^	0.15^c^	0.10	-0.02	0.25^c^	0.25^c^	0.28^c^	0.23^c^	0.05	<0.01	0.05	0.09^d^
Civic engagement	0.39^c^	0.13^c^	0.40^c^	0.63^c^	0.51^c^	0.35^c^	0.46^c^	0.73^c^	0.33^c^	0.11	0.29^c^	0.59^c^
Influence of CHWs on empowerment	0.23^c^	0.12^b^	0.27^c^	0.31^c^	0.77^c^	0.67^c^	0.88^c^	0.75^c^	0.22^c^	0.30^c^	0.20^b^	0.17^b^
Education:												
None	(ref)	(ref)	(ref)	(ref)	(ref)	(ref)	(ref)	(ref)	(ref)	(ref)	(ref)	(ref)
Primary	0.04	<0.01	0.08	0.04	0.11^b^	0.09^a^	0.12^b^	0.11^a^	0.34^c^	0.20^d^	0.46^c^	0.36^b^
Secondary or more	0.14^b^	0.12^b^	0.22^c^	0.09^d^	0.15^b^	0.11^d^	0.17^b^	0.17^b^	0.32^b^	0.14	0.45^c^	0.37^b^
Number of CHW interactions§:												
1 time	(ref)	(ref)	(ref)	(ref)	(ref)	(ref)	(ref)	(ref)	(ref)	(ref)	(ref)	(ref)
2 or 3 times	0.01	0.06^d^	-0.05	0.03	-0.03	-0.08	-0.01	-0.01	-0.02	-0.09	<0.01	0.03
4 or more times	0.09	0.13^b^	0.07	0.06	0.09^d^	0.10^d^	0.12^a^	0.04	0.10^d^	-0.08	0.15^d^	0.22^b^
Satisfied with CHW service	0.04	0.20^c^	<0.01	-0.08	0.25^c^	0.29^c^	0.22^b^	0.25^c^	0.13^a^	0.27^c^	0.14^d^	-0.01

CE-CHS – Client Empowerment in Community Health Systems, CHW – community health workers

*Adjusted for age, education, wealth, geographic location, parity (Bangladesh only), and sex (Haiti only). Statistics: ^a^*P* < 0.05, ^b^*P* < 0.01, ^c^*P* < 0.001, ^d^*P* < 0.1

†All analyses in Bangladesh and Kenya are also adjusted for CHW clustering.

‡For Trust in CHWs Scale, in Bangladesh, limited to only those who had contact with a CHW (n = 1017).

§Adjusted for trust.

Increased trust in CHWs was significantly associated with greater client empowerment in Haiti for the CE-CHS Scale and each of the sub-scales (all *P* < 0.001), and in Bangladesh for the CE-CHS (*P* < 0.1) and *Personal agency around health* sub-scale (*P* < 0.001). Civic Engagement was associated with greater empowerment in all three countries (all *P* < 0.001, except *Personal agency around health* in Kenya, which was non-significant). Increased perceived influence of CHWs on empowerment was significantly associated with higher empowerment in all three countries (most *P* < 0.001). Those with secondary education or higher had significantly higher empowerment than those who had no education in all three countries, albeit with varying levels of significance for sub-scales. Having more CHW interactions was also significantly associated with greater empowerment in all three countries with varying levels of significance. Finally, satisfaction with CHW services was significantly associated with greater empowerment in all three countries, although not for all sub-dimensions in Bangladesh and Kenya, nor for the full scale in Bangladesh.

While the scale performed well overall, we recommend minor changes to how three questions are phrased in future scale implementations, to further strengthen the scale. Study team members from all three countries reviewed these changes to ensure face validity. We recommend that the item “I feel confident sharing health information with my family/friends in one-on-one conversations” be changed to “I feel confident sharing about my health experiences with my family/friends.” Additionally, we recommend that the item “I feel confident sharing health information with my family/friends when we are in a group,” be changed to “I feel confident sharing health information with my family/friends.” These changes help further emphasize that the first item is about sharing personal experiences with clients’ own experiences of health care, while the second item is about sharing health information or knowledge more broadly with their community including family and friends. Additionally, we recommend changing the item “I can participate in making decisions for my community” to “I can participate in making decisions that can improve health in my community,” so that the item more explicitly relates to decisions around community health. **Box 1** includes the final recommended scale items and scoring for the CE-CHS.

Box 1 **Final CE-CHS Scale and scoring**

Now I will read a series of statements. Please let me know how much you agree or disagree with each of these statements by responding that you ‘strongly disagree’, ‘disagree’, ‘agree’, or ‘strongly agree’.

**Personal agency around health:**

1. I feel in control of my health.
2. I know what to do when I have a health problem.
3. I know what to do when there is a health problem in my family.
4. I believe that I can participate in finding a solution to my health problem.
5. When a health problem arises in my family, I am able to help find a solution.
6. When I have a health problem, I advocate for myself to make sure I get good care.
7. When there is a health problem in my family, I advocate for them to get good care.

**Agency in sharing health information with others.**

1. I feel confident sharing about my health experiences with my family/friends.
2. I feel confident sharing health information with my family/friends.
3. I feel confident sharing health information with others in my community in one-on-one conversations.
4. I feel confident sharing health information with others in my community when in group/public settings.

**Engagement with community health systems:**

1. There are ways for me to participate in sharing my concerns/giving feedback to providers/managers at health facilities.
2. There are ways for me to participate in sharing my concerns/giving feedback to local leaders in my community.
3. Most facility providers/managers would listen to any concerns I raise.
4. Most local leaders in my community would listen to any concerns I raise.
5. I can participate in making decisions that can improve health in my community.

**Scoring the Client Empowerment in Community Health Systems (CE-CHS):**

Responses are scored as follows: 1 (strongly disagree), 2 (disagree), 3 (agree), 4 (strongly agree).

Generate mean sub-scale and full-scale scores by taking the mean of non-missing items for a final range of 1 to 4.

## DISCUSSION

The 16-item CE-CHS Scale which contains three sub-scales – *Personal agency around health (*individual-level), *Agency in sharing health information with others (*interpersonal-level), and *Engagement in community health systems* (community-level) – is the first multidimensional scale that measures empowerment specifically in the context of community health. We found strong support for the scale’s validity and reliability across three unique settings. Empowerment, a key process and outcome itself, is recognized as a critical component to institutionalizing community health [12,30,31]. The 16-item CE-CHS Scale or any of its sub-scales, can be used by program managers and governments to measure changes in dimensions of client empowerment over time, including in response to interventions or policy changes.

Consistent with the literature, EFA/CFA confirmed that client empowerment is a multidimensional construct and should be measured as such [32,33]. There were notable differences in levels of the empowerment sub-dimensions by country, which highlights the role of the macrosystem in shaping empowerment. For example, there were lower levels of individual empowerment in Haiti – possibly attributed to prevailing insecurity and bursts of political instability, and slightly higher levels of individual empowerment in Bangladesh and Kenya [34]. There were lower levels of interpersonal and community empowerment in Bangladesh compared to in Kenya and Haiti. It is possible that interpersonal and community empowerment in the context of a family planning-focused survey in Bangladesh is inherently different than in the contexts of general health and maternal and child health in Haiti and Kenya. In Bangladesh, there is not one cohesive community health system, perhaps making interpersonal and community empowerment – for example advocacy within this system – more difficult. Overall, macrosystem factors such as a country’s economic development status, political climate, and access to technology may contribute to differing levels of empowerment at the individual, interpersonal, and community levels. These differences underline the importance of measuring these various levels of empowerment that align with the socioecological model, to better understand which dimensions can be strengthened through programs.

Convergent validity results were quite consistent across the three countries for both the full-scale and sub-scales, suggesting that the three dimensions measured are important components of client empowerment in various settings. Results from this study align with prior literature suggesting that completing more education is associated with greater agency and empowerment [35]. We found that (controlling for trust in CHWs) more CHW interactions was associated with increased empowerment, suggesting that CHW programs are operating as intended – to build relationships and empower communities to enable better health and well-being. Further, we found that service satisfaction was associated with the full CE-CHS scale in Haiti and Kenya, and with the *Personal agency around health* sub-scale in all three countries.

Notably, in Bangladesh and Haiti, client empowerment was associated with trust in CHWs – a scale also developed under the Frontline Health project [29]. Trust in CHWs and client empowerment are inextricably linked and should be measured together, since both are essential for effective community health systems [36-39]. In Kenya, further research with a larger sample size may be needed to elucidate the relationship between these two constructs. Additionally, across all three contexts, positive associations between client empowerment and civic engagement more broadly suggests utility in jointly applying these measures to assess valuable non-health outcomes of community health systems and CHW performance in particular [40].

While not the focus of this paper, it is important to highlight the new, unidimensional 5-item Influence of CHWs on Empowerment Scale. This scale directly and concisely assesses clients’ perceived effect of CHWs on five health-related empowerment behaviors, which align with the personal, interpersonal, and community empowerment sub-dimensions of the CE-CHS Scale. This shorter scale could be included alongside the CE-CHS or given its brevity, could be used on its own to monitor quality improvement of CHW programs and accountability mechanisms for community health (eg, through national surveys and research studies). Implementing both scales together would be valuable for understanding client empowerment not directly related to CHWs, as in the CE-CHS, and for understanding more directly the effect of CHWs on empowerment, as in the Influence of CHWs on Empowerment Scale. Further, implementing these scales in combination with other metrics such as the Multi-dimensional Motivation Scale (also developed under the Frontline Health project), can contribute to measuring progress towards recommendations outlined in the World Health Organization’s CHW Guidelines, the Community Health Roadmap, the Community Health Worker Assessment and Improvement Matrix (CHW AIM) toolkit, and the Minimum Quality Standards and Indicators for Community Engagement [41-45].

This study has several limitations. First, we could not assess predictive validity, as is ideal for scale validation, since we did not have longitudinal data nor enough suitable outcome variables (eg, clients’ health outcomes/service uptake) [19]. We suggest that future studies seek to establish predictive validity of this scale in relation to specific health outcomes of interest. Second, there was some lack of variation in item response frequencies, especially in Haiti. While this is a common phenomenon, and not overly concerning from a psychometric perspective, it still would be preferable to have greater variation across response options [19]. It may be that future implementation will capture such diversity in other contexts. Third, we did not have sufficient statistical power to test for scale invariance by potential subgroup differences. Fourth, non-random sampling approaches were used in Bangladesh and Kenya, thus potentially introducing selection bias to our results. Finally, findings may not be generalizable to other locations within the countries included in this study, other countries, other populations (eg, men), or other community health areas (eg, infectious diseases). These all are rich areas for future research.

## CONCLUSIONS

Findings suggest that the CE-CHS Scale is valid and reliable in varying geographies. The CE-CHS Scale along with the Influence of CHWs on Empowerment Scale and Trust in CHWs Scale can be used in the context of implementation research and interventions focused on building empowerment and trust, such as community women’s groups, community score cards, and community dialogue to build social accountability [7,29,46,47]. We hope researchers and program staff alike will find the CE-CHS Scale useful in assessing client empowerment in the context of community health systems worldwide.
